# Nicotine and Microvascular Responses in Skeletal Muscle from Acute Exposure to Cigarettes and Vaping

**DOI:** 10.3390/ijms241210208

**Published:** 2023-06-16

**Authors:** Christopher R. Pitzer, Eiman A. Aboaziza, Juliana M. O’Reilly, W. Kyle Mandler, I. Mark Olfert

**Affiliations:** 1 Division of Exercise Physiology, School of Medicine, West Virginia University, Morgantown, WV 26506, USA; cpitzer1@uthsc.edu (C.R.P.);; 2 West Virginia Clinical and Translational Science Institute, Morgantown, WV 26506, USA; 3 Center for Inhalation Toxicology, West Virginia University, Morgantown, WV 26506, USA; 4 Department of Physiology, Pharmacology and Toxicology, West Virginia University, Morgantown, WV 26506, USA

**Keywords:** 3R4F reference cigarette, e-liquid, intravital microscopy, electronic cigarette, E-cigarette

## Abstract

Despite claims of safety or harm reduction for electronic cigarettes (E-cig) use (also known as vaping), emerging evidence indicates that E-cigs are not likely safe, or necessarily safer than traditional cigarettes, when considering the user’s risk of developing vascular dysfunction/disease. E-cigs are different from regular cigarettes in that E-cig devices are highly customizable, and users can change the e-liquid composition (such as the base solution, flavors, and nicotine level). Since the effects of E-cigs on the microvascular responses in skeletal muscle are poorly understood, we used intravital microscopy with an acute (one-time 10 puff) exposure paradigm to evaluate the individual components of e-liquid on vascular tone and endothelial function in the arterioles of the gluteus maximus muscle of anesthetized C57Bl/6 mice. Consistent with the molecular responses seen with endothelial cells, we found that the peripheral vasoconstriction response was similar between mice exposed to E-cig aerosol or cigarette smoke (i.e., 3R4F reference cigarette); this response was not nicotine dependent, and endothelial cell-mediated vasodilation was not altered within this acute exposure paradigm. We also report that, regardless of the base solution component [i.e., vegetable glycerin (VG)-only or propylene glycol (PG)-only], the vasoconstriction responses were the same in mice with inhalation exposure to 3R4F cigarette smoke or E-cig aerosol. Key findings from this work reveal that some component other than nicotine, in inhaled smoke or aerosol, is responsible for triggering peripheral vasoconstriction in skeletal muscle, and that regardless of one’s preference for an E-cig base solution composition (i.e., ratio of VG-to-PG), the acute physiological response to blood vessels appears to be the same. The data suggest that vaping is not likely to be ‘safer’ than smoking towards blood vessels and can be expected to produce and/or result in the same adverse vascular health outcomes associated with smoking cigarettes.

## 1. Introduction

Microvasculature is intimately linked to organismal health due to the role that blood vessels play in providing oxygen and nutrients, transporting carbon dioxide and metabolic waste, and acting as a conduit for cellular communication. Given the importance of the vascular system, it is not surprising that the remodeling or dysfunction of blood vessels directly correlates with early senescence and disease outcomes [1,2]. The negative effects of cigarette smoking on the vascular system are well established and include atherosclerosis, coronary heart disease, aneurism, stroke, peripheral arterial disease, and microvascular dysfunction (see reviews [3,4,5]).

Novel tobacco products, such as heated tobacco products (e.g., IQOS) and electronic cigarettes (E-cigs) have gained prominence over the last 15 years, with the global E-cig market calculated to be worth ~15 billion U.S. dollars in 2020 and expected to grow by 5 times that value by 2028 [6]. The rapid growth of the E-cig market is fueled by marketing and claims that E-cigs are a safer alternative to traditional smoking [7], yet much remains unknown about the health effects of E-cigs, especially in terms of their impact on microvascular health. Claims of safety or harm reduction are based, in part, on evidence that the aerosol cloud from E-cigs often contains fewer chemical compounds than the smoke from traditional cigarettes [8]. However, it has long been understood (dating back to Paracelsus, 1493–1541) that it is the ‘dose that makes the poison’, and toxicity is not simply equivalent to the number of chemicals [9]. Despite variations in the physical appearance and operational settings among brands (or types) of E-cigs, these devices all function to heat a ‘base solution’. The base solution (also known as e-liquid or e-juice) typically comprises a mixture of propylene glycol (PG) and vegetable glycerin (VG), to which flavorings and nicotine are usually added according to the users’ preference [10,11]. Some E-cig devices (e.g., third generation tank-style ‘mod’ E-cigs) also allow users to control the voltage or heating temperature, which in turn can significantly alter the chemical profile of the vape cloud that is inhaled [12,13]. Thus, the toxicity potential to human health becomes multifaceted and more complex compared to traditional cigarettes because users can change the nicotine concentration [14] or composition of the base solution [15], add flavors (i.e., adding more chemicals) [16,17], and in some devices, even change the heating temperature. The latter can significantly alter the chemical profile of the cloud, even if all the other conditions are kept consistent [18].

While the long-term health outcomes to humans from novel tobacco products are yet to be determined, acute studies in animals and humans suggest that E-cigs have similar, if not the same, effects on vascular function as traditional cigarettes [19]. We, and others, have reported that E-cig exposure impairs vascular endothelial cell function and increases arterial stiffness in animals [19,20] and humans [21,22]. However, the contribution from the individual components of E-cig liquid is still poorly understood. This is important because users select the composition of the e-liquid they use; therefore, understanding the potential effects of these components on microvascular health and function is vital before claims of safety or harm reduction are touted.

Given the dynamic response and importance of the skeletal muscle vascular bed in health and disease, the purpose of this study was to evaluate the contribution of the base components of E-cig liquid on in vivo vascular function in skeletal muscle using an intravital microscopy approach. This approach allows the direct visualization of arterioles to assess both the acute effects on vascular tone and the endothelium-dependent-dilation (EDD) responses. Vessel tone and EDD in this vascular bed are important because arterioles serve as the primary site of resistance in the cardiovascular system, and are critically important for maintaining healthy blood pressure and the supply of nutrients to downstream capillaries. We hypothesized that if E-cigs are safer than traditional cigarettes, then the physiological and molecular response of blood vessels and endothelial cells to E-cig aerosol exposure will be different than that observed from cigarette smoke.

## 2. Results

**Figure 1:**
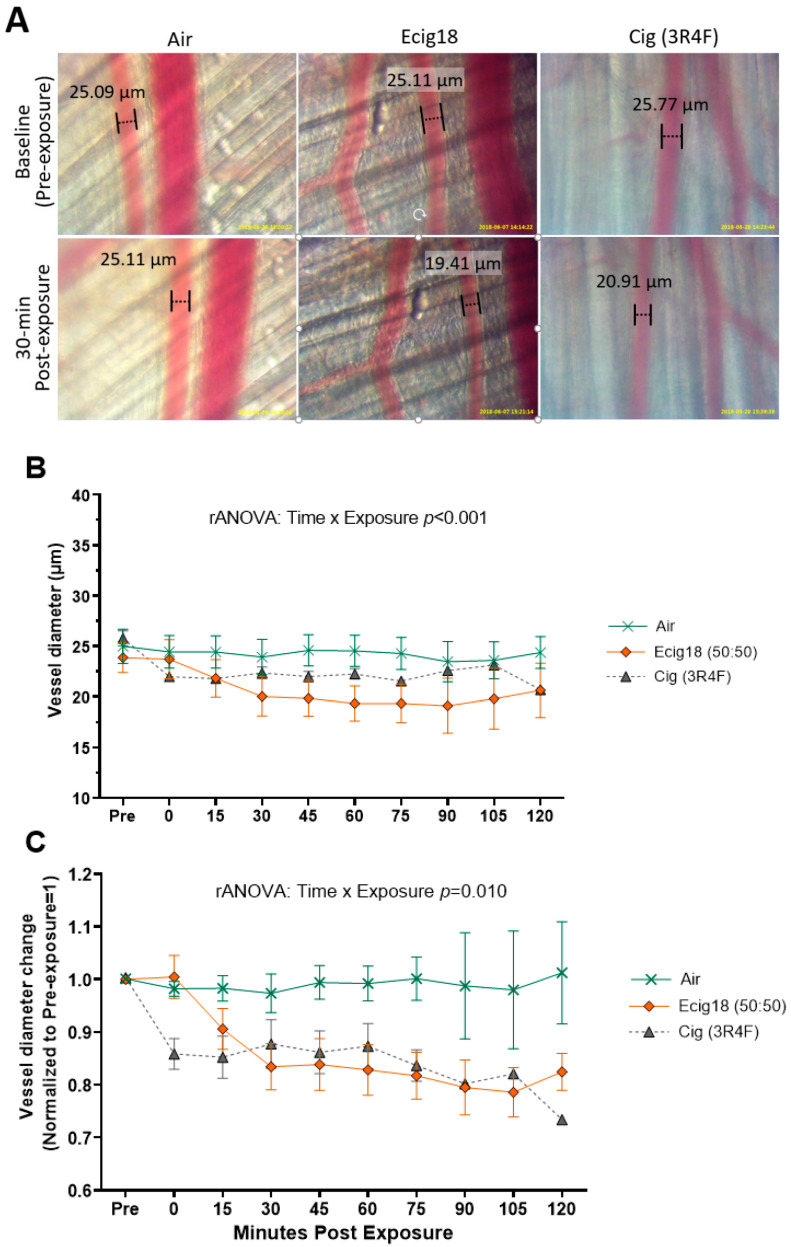
Two-hour time course comparisons of second-order arteriole skeletal muscle (gluteus maximus m.) responses to inhalation exposure to air, 3R4F reference cigarette, or electronic cigarettes (E-cig) aerosol. (**A**) Visual of representative vessels from intravital imaging (20× objective) from each exposure group at baseline and 30 min post-exposure. Arteriole diameters measurements are included in the images for reference. (**B**) Group averaged arteriole diameters, and (**C**) arteriole dimeters normalized to pre-exposure (i.e., showing change from baseline set at 1.0). The number of animals in each group is as follows: Air (*n* = 10), Cig 3R4F (*n* = 6), E-cig18 (*n* = 12).

In our first animal cohort, we observed the vascular tone of second-order arterioles for up to 120 min in mice acutely exposed to either room air (sham exposure) (*n* = 10), E-cig (containing 50:50 VG:PG with 18 mg/mL nicotine and French vanilla flavor, Ecig-18) (*n* = 12), or 3R4F reference cigarettes (Cig, *n* = 6). Repeated measures (rANOVA) analysis showed a significant exposure × time interaction with respect to the vessel diameter (*p* < 0.001) (Figure 1A,B), and the vessel diameter normalized to its starting diameter (i.e., percentage change from baseline, *p* = 0.01) (Figure 1C). Vasoconstriction reached its nadir 15–30 min after exposure and remained constricted for >2 hour. E-cig and 3R4F cigarette showed a similar magnitude of vasoconstriction 15 to 120 min post exposure.

**Figure 2:**
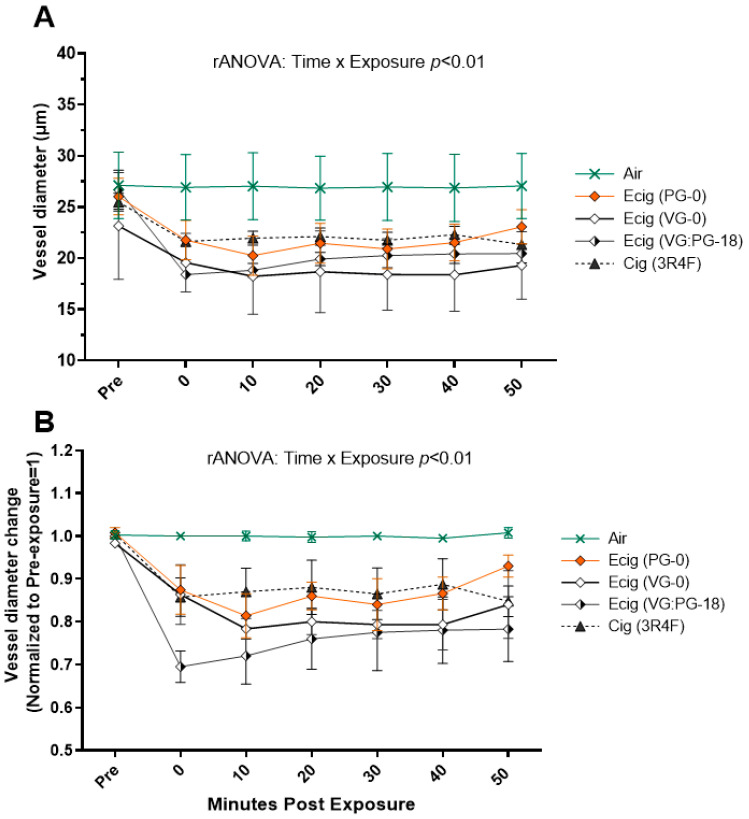
(**A**) One-hour time course of second-order arteriole diameter and (**B**) change in arteriole diameters normalized to pre-exposure (baseline set at 1.0) in mice with inhalation exposure (10 puffs over 5 min) to Air, 3R4F reference cigarette, and E-cig aerosol created from different e-liquid compositions (i.e., 100% propylene glycol with no nicotine and no flavor, PG-0; 100% vegetable glycerin with no nicotine and no flavor, VG-0; and VG:PG (50:50 by volume) with 18 mg nicotine with French vanilla flavor, VG:PG-18). The number of animals in each group were as follows: Air (*n* = 5), PG-0 (*n* = 3), VG-0 (*n* = 3), VG/PG-18 (*n* = 10), and 3R4F (*n* = 5).

In the second animal cohort, we characterized the contribution of different components of e-liquid to the observed vasoconstriction by performing exposure using Ecig with VG-only (with no nicotine and no flavor, VG-0) (*n* = 3); PG-only (with no nicotine and no flavor, PG-0) (*n* = 3); and 50:50 VG:PG with 18 mg/mL nicotine with French vanilla flavor (VG/PG-18) (*n* = 10) and 3R4F reference cigarettes (Cig) (*n* = 5) (Figure 2). As seen in Figure 2, at all timepoints, the absolute and normalized vessel diameter responses were similar for Ecigs with VG-0, PG-0, or VG/PG-18 compared to the 3R4F reference cigarette, and all E-cig exposure groups had reduced arteriole diameters compared to the air-exposed animals (*n* = 5) (exposure x time interaction, *p* < 0.01).

**Figure 3:**
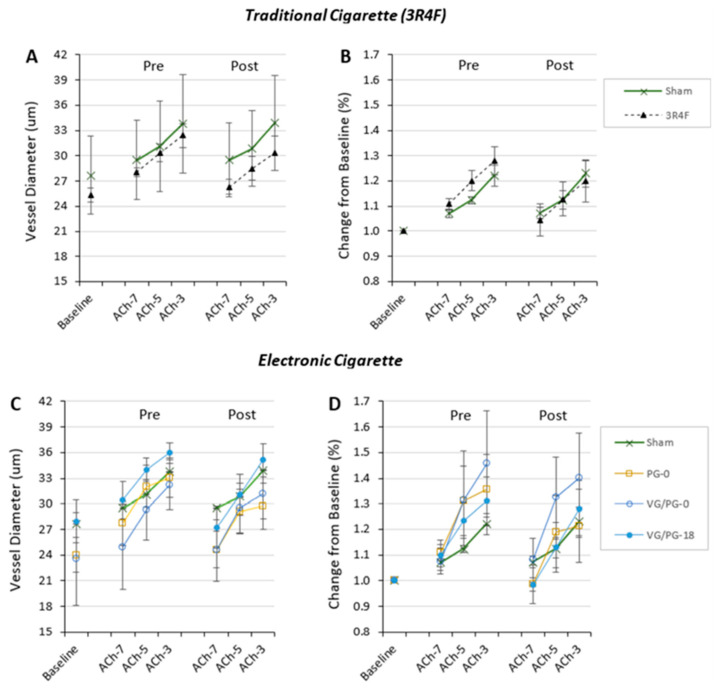
Arteriolar diameter at baseline and dose response to acetylcholine (ACh from 10^−7^ to 10^−3^ M) before and after inhalation exposure (10 puffs over 5 min) shown in Figure 2. Aortic reactivity showing (**A**) vessel diameter and (**B**) change in diameter normalized to baseline for exposure to 3R4F cigarette smoke is shown in Panels A and B. Aortic reactivity showing (**C**) vessel diameter and (**D**) change in diameter normalized to baseline for exposure to E-cig aerosol from different e-liquid composition is shown in C and D. (i.e., 100% propylene glycol with no nicotine and no flavor, PG-0; 100% vegetable glycerin with no nicotine and no flavor, VG-0; VG:PG (50:50 by volume) with 18 mg nicotine with French vanilla flavor, VG:PG-18). The number of animals in each group were as follows: Sham=Air exposed (*n* = 3), PG-0 (*n* = 6), VG/PG-0 (*n* = 3), VG/PG-18 (*n* = 4), and 3R4F (*n* = 5).

The endothelial cell-mediated vasoreactivity of the arterioles was also assessed using acetylcholine (ACh) before and 60 min after exposure (i.e., EDD). Figure 3 shows similar changes in the absolute and normalized vessel diameters, and we found no difference in EDD reactivity before or after exposure in either the 3R4F- (*n* = 5) (Figure 3A,B) or E-cig- exposed groups (Figure 3C,D) (number of animals in each group are as follows; Sham *n* = 3, PG-0 *n* = 6, VG/PG-0 *n* = 3, VG/PG-18 *n* = 4).

## 3. Discussion

The main findings of this work are the following: (1) arteriole responses in a peripheral vascular bed respond rapidly (within 15 min) to the inhalation of cigarette smoke and E-cig aerosol, and (2) that the vascular responses are similar between cigarettes (i.e., 3R4F) and E-cigs. While the scope of this observational study was not designed to uncover cellular or molecular mechanisms, an important finding from our work is that the peripheral skeletal muscle vasoconstriction response triggered by E-cigs is not mediated by nicotine, suggesting that vasculopathies related to the chronic inhalation of vape and/or cigarette smoke exposure are likely due to other compounds/chemicals from the delivery vehicle (constituents of smoke from cigarettes and base e-liquid for E-cigs, i.e, VG and PG). Indeed, there are several classes of chemicals produced in both cigarette smoke and E-cig aerosols, which include, but are not limited to, volatile organic compounds, polycyclic aromatic hydrocarbons, carbonyls, and heavy metals [23,24,25,26]. Carbonyl compounds and reactive aldehydes (such are formaldehyde, acetaldehyde, acrolein, etc.) may be of particular interest since recent evidence has found that they can also influence vascular function [27,28]. Evidence of heavy metals (such as nickel, cadmium, copper, lead, and others) has likewise been reported in cigarette smoke and E-cig aerosol [29,30,31] and, in some cases, these metals have been found to impair vascular function [32]. Whether these, and/or other compounds, are responsible for the effects we see here are still unknown, and requires a more in-depth study and investigation.

### 3.1. Nicotine

Nicotine is a sympathomimetic drug which can stimulate increases in heart rate, cardiac contraction, and blood pressure by acting on nicotinic acetylcholine receptors (nAChRs). While there is an abundance of data reporting that nicotine is capable of constricting blood vessels via several mechanisms (see review [33]), it is important to note that much of this evidence comes from studies that have used other (than inhalational) methods for nicotine delivery, such as intravenous, transdermal, or oral routes (i.e., gum, medication, etc.) [34,35,36,37]. Indeed, intravital and ex vivo vascular studies that topically applied or intravenously delivered nicotine do observe vasoconstriction [38,39]. Until the advent of E-cigs, very few studies had used inhaled or aerosolized nicotine, or evaluated the vehicle aerosol with and without nicotine on vascular effects. The customizable nature of E-cigs and their e-liquid provides the ideal opportunity to independently assess the effect of the delivery vehicle with and without nicotine. In contrast to the established dogma, our data suggest that much, if not all, of the vasoconstriction occurring due to acute inhalation exposure is not likely due to nicotine. This agrees with recent data from our group that also shows that the offspring of rodent dams exposed to E-cig aerosol while in utero have vascular endothelial dysfunction independent of the presence of nicotine due to maternal vaping during pregnancy [40,41]. The rescue of EDD in these offspring by incubation with inhibitors of oxidative stress (such as tempol and febuxostat) indicates the role of reactive oxygen species (ROS) and a reduction of nitric oxide bioavailability, a mechanism likely to be involved in the peripheral vasoconstriction we report after acute exposure [42,43]. Given the similarity of the vascular responses seen with cigarette smoke and E-cigs, it is reasonable to think that the same mechanism(s) are involved for smoking too. This is supported by recent molecular evidence from in vitro studies showing that eNOS activation is similarly impaired in people using E-cigs and cigarettes compared to never users/smokers [44]. Moreover, a progressive (dose-dependent) increase in cell death was seen in the endothelial cells with the increasing concentration of the e-liquid vehicle (i.e., 0.0001–1% dilutions of PG:VG), with and without nicotine [44]. These molecular responses are consistent with the evidence we report here, demonstrating that nicotine is not a major or primary contributor to the vascular dysfunction occurring from inhalation exposures. At present, we do not know which chemicals or compounds are responsible, as the literature illustrates that there are many chemicals/toxins common to both cigarette smoke and vape clouds [23,24,25,26]. Some of these (i.e., formaldehyde and acetaldehyde) are also reported in the etiology of endothelial cell dysfunction [27,28], but it is possible that different toxicants and other mechanisms may be triggered by E-cig aerosol and cigarette smoke. One possibility may also involve airway irritant receptors triggering neural feedback mechanisms. Recent evidence has found that airway irritant receptors trigger neural feedback mechanisms, and that bilateral vagotomy prevents endothelial dysfunction induced by acute exposure to E-cigs and cigarettes [45]. Regardless of the etiology, these data are consistent with emerging evidence showing comparable acute impairments in vascular function across a wide range of E-cigs devices with and without nicotine [46].

It is important to emphasize that we are not suggesting that nicotine has no influence or effect on the cardiovascular system (see review [47]). Indeed, recent preclinical studies in mice creating aerosolized nicotine by bubbling free-base nicotine with air (at a concentration similar to smokers and vapers) have found changes in blood pressure and cardiac remodeling [48,49]. Moreover, evidence from animal embryo models has revealed the greater incidence and severity of cardiac defects with nicotine exposure with E-cig aerosol or cigarette smoke extracts [50]. Thus, while nicotine is indeed a sympathomimetic drug with influences on cardiac and hemodynamic function, our data provide clear evidence that the vasoconstriction of peripheral arterioles in skeletal muscle after E-cig exposure occurs independent of nicotine.

### 3.2. E-Liquid Base Solution

The composition of e-liquid (i.e., ratio of VG to PG) varies based on users’ preferences and/or manufacturers’ product lines. Advanced or experienced users develop preferences and even custom recipes for VG and PG, which are discussed in vaping-related online forums [51]. These preferences are linked to different airway sensory effects created by VG and PG, which are associated to user satisfaction [52]. Although both VG and PG are humectants that are Generally-Recognized-As-Safe (G.R.A.S.) by the US Food and Drug Administration, it is important to note that this designation only applies to topical and oral ingestion. Indeed, both compounds are widely used in products around the world (e.g., shampoo, skincare products, ketchup, oral medications, etc.). It is interesting to note, even before E-cigs were widely available for recreational use in North American and European markets (early 2007), that there were early indications from the entertainment industry that mineral oils and glycols (e.g., VG and PG used in ‘fog-machines’) were associated with adverse respiratory symptoms, such as wheezing, coughing, dry mouth, and chest tightness) [53]. Our study extends this knowledge to the vascular effects from acute exposure, and also provides clear evidence that exposure to nicotine-free PG and VG (i.e., PG-0, VG-0), even without flavorings, trigger vasoconstriction in peripheral arterioles. Our data show that either VG or PG can equally influence vascular tone, and both (VG and PG) trigger responses that are similar to that observed from cigarette smoke.

It is notable that we do not observe any difference in the EDD response to acetylcholine with the current exposure paradigm (i.e., one-time acute exposure to 10 puffs used here), whereas other studies have shown impairments in EDD after exposing rodents to an exposure similar to ours with a variety of E-cig devices, including ultrasonic E-cig devices and JUUL E-cigs (e.g., pod-style E-cigs) [54]. There is also evidence in the literature showing that flow-mediated dilation (FMD), an integrated vascular response to hyperemic-induced shear stress, is equally impaired by traditional cigarettes, E-cigs [46], and IQOS (i.e., heat-not-burn cigarettes) [55]. Likewise, smoking is associated with the dose-related impairment of FMD and increased intima-media thickness (IMT) of the carotid arteries [20,56], and we have previously reported that chronic exposure to both 3R4F smoke and E-cig vaping results in the similar stiffening of the arteries in mice [19]. One explanation for the discrepancy in our EDD responses compared to others is that we delivered ACh directly into the water bath; thus, we may have triggered extravascular pathways (independent of endothelial cells) for NO production to cause the vasodilation we observed. The totality of our exposure was also only 10 puffs to an animal that had never been exposed before, so it may also be that a greater exposure dose is required to impair EDD reactivity. Nonetheless, given the overall similarity in physiological responses with cigarettes and E-cigs that we report here, and noted in the literature (cited above), it seems improbable that E-cigs are (or will prove to be) ‘safer’ than cigarettes in the context of microvascular function and the cardiovascular diseases associated with their use.

### 3.3. Flavors

There is ample evidence that flavors added to e-liquid can increase the toxicity of the E-cig aerosol [16,57,58,59]. Although our study was not designed to evaluate the effects of flavorings *per se*, we did have exposures where the e-liquid contained vanilla flavoring (i.e., VG/PG-18 exposure). While vanillin (and other flavors) has been shown to impair endothelial cell nitric oxide synthesis in vitro [60], we did not see a significant difference in the vascular response to E-cig aerosol with e-liquid that contained vanilla-flavoring (VG/PG-18) compared to unflavored e-liquid (VG-0 or PG-0)(as seen in Figure 2 and Figure 3). There is, however, an extensive range of flavors (i.e., >10,000) and some flavors are known (when aerosolized) to produce toxic compounds that have significant adverse biological and health effects [17,58,60,61,62]. Thus, while the vanilla-based flavor under the acute exposure conditions we used did not result in a measurable vascular effect in our study, this would not preclude other flavors from having very different outcomes.

### 3.4. Study Limitations

There are limitations to this work that should be noted. First, because these data are from an animal model, a direct correlation to humans must be viewed with caution. However, we also note that these data agree with an emerging body of human literature with E-cigs, and in the context of smoking (with nearly a century of research involving rodents and human cigarette exposures), rodent models of inhalation exposures have proven to be a good predictor of adverse health outcomes. The novelty and importance of this study stems from the physiological evaluation of the acute effects of the individual components of the e-liquid used in E-cigs and a direct comparison to cigarette smoke. Another limitation in our study is that our sample size was small. However, since we can compare the post-exposure vessel diameter to the baseline for each animal, each animal can serve as their own control, which provides clinical relevance and allows for greater statistical power (with a smaller number of animals) than if these studies relied on a cross-sectional study design. Moreover, the lack of blood analyses for nicotine (or cotinine) levels between the 3R4F and E-cig group (i.e., VG:PG-18) containing nicotine means that we do not know if the 3R4F cigarette and VG:PG-18 groups had similar circulation nicotine levels. Nevertheless, given that the study design included a comparison to no nicotine e-liquid/aerosol, and that our overall finding was that vascular dysfunction was similar in the presence or absence of nicotine, our overall interpretation is neither confounded nor altered by not knowing the circulating nicotine concentration for those exposed to nicotine.

## 4. Materials and Methods

### 4.1. Animals

All experiments were performed in compliance and with oversight by the West Virginia University Institutional Animal Care and Use Committee. Eight- to ten-week-old C57BL/6 mice were obtained from Jackson Laboratories (Bar Harbor, ME, USA) and housed in a temperature- and humidity-controlled pathogen-free vivarium. The animals were kept under a 12 h light/dark cycle and provided ad libitum access to standard rodent food and water. Mice were studied at ~12 weeks (3 months) of age.

### 4.2. Intravital Microscopy 

Mice were anesthetized via intraperitoneal injections of 100 mg/kg of thiobutabarbital (Inactin). After the depth of anesthesia was determined to be appropriate, an endotracheal tube (PE90 polyethylene tubing) was inserted via tracheostomy, sutured in place, and trimmed below the chin to mimic the similar anatomical dead space of the respiratory system above the trach insertion site. Thereafter, a superficial incision was made above the gluteus maximus tendon (slightly lateral to the dorsal midline). The gluteus maximus was carefully dissected away from the gluteus medius, while the surgical area was kept moist using normal saline as needed throughout the surgery. Traction was applied to the muscle using silk sutures tied through the tendon to maintain shape as the muscle was submerged in physiological saline solution in a custom-built intravital microscopy stage. “Double loop” knots were used to prevent the suture from applying pressure to the muscle. Microscope evaluation (using 20× water immersion lens) was used to visualize the second-order arterioles within the muscle. Heart rate and body temperature were monitored throughout the entire procedure, and body temperature was maintained using a thermally regulated stage receiving input from a rectal thermometer.

The muscle remained submerged in a physiological saline solution (PSS; 119 mM NaCl, 25 mM NaHCO_3_, 6 mM KCl, pH 7.35–7.40) bubbled with 5% carbon dioxide and warmed to 37 °C. PSS was continually perfused into the chamber containing the externalized gluteus maximus at a rate of 4 mL/min to ensure that fresh solution was present and to maintain bath temperature. Images of arterioles were collected at predetermined timepoints, and the vessel diameter was measured using ImageJ v1.51 software. ImageJ measurements were scaled by calibrating software using a 0.01 mm microscope micrometer on a glass slide (where the scale was determined to be 300 pixels = 100 um). The vessel diameter was reported as the average of three separate measurements of the same vessel from different areas of a single image.

### 4.3. Inhalation Exposure

After intravital surgery prep was completed, the externalized muscle was allowed to equilibrate in the water bath for 15 min. Thereafter, a baseline image of the selected arteriole was captured, then by random selection, the animal was exposed (via blow-by to tracheotomy) to 10 puffs of cigarette smoke, E-cig aerosol, or ambient air. Cigarette and E-cig exposure occurred over 5 min with puffs given every 30 s. A rodent ventilator (Harvard Apparatus) was used to generate 55 mL of puff volumes and tubing was used to direct the smoke/aerosol to the tracheostomy tube. Cigarette smoke was generated using 3R4F reference cigarettes (Univ. of Kentucky), and for E-cig aerosol, we used a commercially available “tank style” E-Cig device with the voltage set to 4.8 V and 17.5 watts (eGrip™OLED, Joyetch, Shenzhen, China). E-liquids were purchased from a local vape shop, and comprised 50:50 VG:PG (French vanilla) with either 0 mg/mL or 18 mg/mL nicotine (VG/PG-0 and VG/PG-18, respectively), VG-only (no nicotine, no flavor, VG-0), or PG-only (no nicotine, no flavor; PG-0).

The purity of the e-liquid composition was based on manufacture labeling and was not independently evaluated, with the exception of the nicotine concentration level, where the absence of nicotine was determined in solutions reporting 0 mg/mL or in those not expected to have nicotine (i.e., VG- or PG-0 only), and 18 mg/mL of nicotine was verified in nicotine-containing e-liquid using HPLC. The composition of the 3R4F reference cigarettes is reported by the University of Kentucky, Center for Tobacco Reference Product on their website [63].

### 4.4. Vascular Assessments

Vascular assessment was made in two different cohorts of mice. In the first cohort, in vivo arteriole diameters were measured at baseline (before any exposure) and immediately after the completion of inhalation exposure. Vessel diameters (i.e., images) were remeasured every 15 min for up to 2 h.

In the second cohort, in vivo arteriole diameters were immediately measured after inhalation and then at 10-min intervals for 50 min. The second cohort included the assessment of arteriole reactivity to acetylcholine (ACh) measures at baseline (before any exposure) and 60 min after exposure. The reactivity of the arterioles to acetylcholine (ACh) was performed using 10^−7^, 10^−5^, and 10^−3^ M ACh added directly into the water bath. ACh was allowed to incubate for 5 min under each concentration before imaging. Data are presented as both the actual vessel diameter over time and vessel diameter as a percentage of the baseline diameter at each experimental timepoint.

### 4.5. Statistical Analysis

Repeated measures ANOVA was used evaluate the multiple time-dependent measurements in the same experiment between the different inhalation exposures. If a significant treatment by time interaction was found, post hoc testing (Student’s *t*-test) was used to identify differences at time points between the exposure groups.

## 5. Conclusions

Although the lungs are the first major organ to encounter inhalation exposure, it is well established that airborne exposures (such as smoking, air pollution, etc.) will negatively affect multiple organ systems, including the microvascular bed. Contrary to the claims by proponents for the tobacco industry and even some public health authorities [64], our preclinical data suggest that there is great cause for concern toward vascular health related to vaping E-cigs. Based on the known risks and adverse vascular outcomes from smoking, combined with the similar acute physiological responses we report here between cigarettes and E-cigs, the only logical conclusion is that E-cigs will lead to the same risk for cardiovascular disease as seen with smoking. This notion is supported by an ever-growing body of literature using different methodologies and exposure models (e.g., [20,21,42,65,66,67]) to demonstrate that vaping and smoking produce similar significant and adverse changes to vascular structure and function. While our data suggest that nicotine is not the culprit in the context of acute vascular dysfunction, we cannot exclude the effects that nicotine may have on other organs and their sequelae. These data should serve as a warning that E-cigs should not be viewed as ‘safer’ than cigarettes, and should ultimately cast doubt on claims that E-cigs are suitable as harm reduction devices.

## Data Availability

Data are available upon request via email to the corresponding author.
